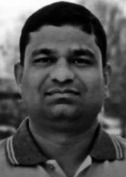# Organomercury Nucleic Acids: Past, Present and Future

**DOI:** 10.1002/cbic.202000821

**Published:** 2021-02-16

**Authors:** Dattatraya Ukale, Tuomas Lönnberg

**Affiliations:** ^1^ Department of Chemistry University of Turku Vatselankatu 2 20014 Turku Finland

**Keywords:** base pairing, coordination, mercury, nucleic acids, organometallic

## Abstract

Synthetic efforts towards nucleosides, nucleotides, oligonucleotides and nucleic acids covalently mercurated at one or more of their base moieties are summarized, followed by a discussion of the proposed, realized and abandoned applications of this unique class of compounds. Special emphasis is given to fields in which active research is ongoing, notably the use of Hg^II^‐mediated base pairing to improve the hybridization properties of oligonucleotide probes. Finally, this minireview attempts to anticipate potential future applications of organomercury nucleic acids.

## Graphical abstract

**The organomercury renaissance**: Research on the synthesis and applications of organomercury nucleic acids and their constituents is reviewed, with a focus on recent developments, such as efforts to improve the hybridization properties of oligonucleotide probes through Hg^II^‐mediated base pairing of covalently mercurated base moieties.

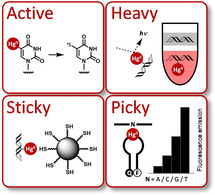

## 1 Introduction

Mercury is one of the metals most extensively studied for its interactions with nucleic acids. In line with the softness of Hg^II^, these interactions mainly take the form of coordination to the nitrogen donors of nucleobases. Mercury also forms fairly stable organometallic compounds, in many cases under conditions withstood by nucleic acids. In fact, natural nucleic acids feature two sites that are readily mercurated by simple treatment with Hg^II^ salts, namely the C5 atoms of cytosine and uracil bases. Appropriately designed artificial nucleobases further widen the range of organomercury modifications that can introduced to oligonucleotides and nucleic acids.

Numerous applications of organomercury nucleic acids and their constituents have been proposed over the past five decades. Some, such as density labeling in pycnographic analysis have only seen limited popularity while others, such as affinity tagging, have been phased out by more modern methods after a brief period of usage. Yet others, notably the utilization of covalently mercurated nucleobases as synthetic intermediates, are still seen occasionally. Finally, organomercury oligonucleotides as probes in single nucleotide polymorphism (SNP) genotyping and mercurated nucleotides as markers in electron microscopy DNA sequencing are examples of promising future applications still in their infancy. This minireview summarizes the synthetic methods towards organomercury nucleic acids and oligonucleotides, outlines their historic and contemporary applications and attempts to shed light on future prospects.

## 2 Synthesis of Organomercury Nucleosides, Nucleotides and Nucleic Acids

### 2.1 Electrophilic aromatic substitution by Hg^II^

**Figure 1:**
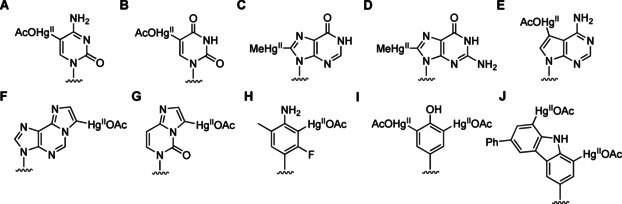
Examples of covalently mercurated natural and artificial nucleobases. In most cases, mercuration has been proven with both monomers and oligonucleotides or nucleic acids.

Mercuration of aromatic rings through electrophilic aromatic substitution has been known since the mid‐1800s.[^1^, ^2^] With unactivated aromatic hydrocarbons prohibitively harsh conditions are required but electron‐rich arenes, including nucleobases, readily react under conditions tolerated by nucleic acids.[^3^, ^4^] The most reactive sites are the C5 atoms of cytosine and uracil, quantitatively mercurated in 2 h with 4 mM aqueous mercuric acetate at pH 6.0 and 50 °C (Figure 1A and B). It should be noted, however, that an excess of mercuric acetate is typically required owing to competing coordinative interactions with the endocyclic nitrogen atoms, especially thymine and uracil N3 and guanine N1.^[5]^ The purine bases are inert to electrophilic aromatic substitution by mercuric acetate under conditions feasible for the mercuration of nucleosides. 8‐Methylmercurypurine nucleosides, however, can be prepared by 30 min treatment with methylmercuric nitrate at pH 7 and 50 °C (Figure 1C and D).^[6]^ Analogously to the reaction of pyrimidine bases and mercuric acetate, covalent mercuration at C8 only takes place once all of the nitrogen donors have been saturated.

Artificial nucleoside and nucleotide analogues with sufficiently electron‐rich base moieties greatly widen the scope of covalent mercuration. C7 of 7‐deazaadenosine‐5′‐triphosphate, for example, can be mercurated under the same conditions as C5 of cytosine and uracil (Figure 1E).^[3]^ With 1,*N* ^6^‐ethenoadenosine, the etheno bridge introduces a new site of covalent mercuration, namely the carbon atom bonded to N1 (Figure 1F).^[7]^ Interestingly, 3,*N* ^4^‐ethenocytidine also undergoes mercuration at the corresponding carbon atom rather than C5 (Figure 1G). Mercuration can be promoted at a desired site by activating *ortho*/*para* directors, such as hydroxy or amino substituents, and prevented at an undesired site by alkyl substituents. Facile and selective mercuration at C2 of a 3‐fluoro‐6‐methylaniline C‐nucleoside (Figure 1H) provides an illustrative example of the power of this approach.^[8]^ Nucleobase analogues with multiple electron‐rich carbon atoms, such as phenol^[9]^ or 6‐phenyl‐1*H*‐carbazole^[10]^ (Figure 1I and J), can be mercurated more than once although at progressively slower rates.

### 2.2 Direct mercuration of oligonucleotides and nucleic acids

Electrophilic aromatic substitution at C5 of cytosine and uracil bases, as well as electron‐rich carbon atoms of artificial nucleoside analogues, by Hg^II^ proceeds under sufficiently mild conditions to be feasible also on oligonucleotides^[11]^ and even longer nucleic acid sequences.^[4]^ The reaction is highly sequence dependent, homopolymers of cytidine and uridine being mercurated much faster than naturally occurring heteropolymers. In contrast, secondary structure of the nucleic acid hardly affects the rate of mercuration, probably because of the denaturing conditions of the reaction mixture.^[4]^ Thiol‐promoted demercuration, on the other hand, can be performed under conditions where the polymercurated nucleic acid retains its secondary and tertiary structure and in such cases site‐dependent reactivity patterns are observed. This selectivity has been harnessed for the preparation of a monomercurated tRNA although homogeneity of the product in terms of the site of mercuration was not established.^[12]^ With synthetic oligonucleotides, preventing off‐target mercuration is straightforward as any cytosines and uracils that should remain unreacted can be replaced with the inert 5‐methylcytosines and thymines, respectively.

### 2.3 Enzymatic polymerization of organomercury nucleotides

The utility of organomercury nucleotides as substrates of polymerases depends strongly on the ligand sphere of Hg^II^. With relatively weakly coordinating ligands, such as acetato or chlorido, organomercury nucleotides are potent inhibitors of both DNA and RNA polymerases, in all likelihood owing to coordination of Hg^II^ to a critical sulfhydryl group.[^3^, ^13^, ^14^] In the presence of a thiol ligand, polymerization proceeds smoothly albeit with some concomitant demercuration.^[15]^ Interestingly, different enzymes exhibit different requirements for the thiol ligand – although 2‐mercaptoethanol is the ligand of choice in most cases, some enzymes, notably calf thymus terminal deoxynucleotidyl transferase, prefer a sterically less demanding ligand such as methane‐ or ethanethiol.^[3]^

Enzymatic polymerization of 5‐mercuripyrimidine nucleotides has been proven with both template‐directed^[16]^ as well as template‐independent^[13]^ polymerases. In the former case, 5‐mercuriuridine‐5′‐triphosphate was readily incorporated on a poly[d(AT)] but not on a poly(dG)⋅poly(dC) template while the opposite is true for 5‐mercuricytidine‐5′‐triphosphate, thus suggesting that the fidelity of the enzymatic reaction is retained. The scope of other modifications tolerated on the organomercury nucleotide remains obscure, as the studies found in the literature are limited to derivatives of natural nucleotides as substrates.

### 2.4 Post‐synthetic introduction of organomercury nucleobase surrogates

As discussed above, direct mercuration of oligonucleotides by electrophilic aromatic substitution is a feasible strategy when the aromatic ring to be mercurated is sufficiently electron‐rich. With less reactive systems, the conditions required can be so harsh that competing reactions, such as RNA cleavage,^[17]^ become a problem. In such cases, the organomercury moiety can first be synthesized separately using whatever conditions necessary and then introduced to the oligonucleotide by suitable conjugation chemistry. The feasibility of this strategy has been demonstrated recently by oximation of a support‐bound aminooxy‐functionalized oligonucleotide with 2‐mercury‐3‐hydroxybenzaldehyde.^[18]^

## 3 Organomercury Nucleosides, Nucleotides and Nucleic Acids as Reactive Intermediates

### 3.1 Palladium‐catalysed cross‐coupling reactions

**Scheme 1:**
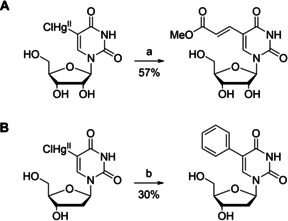
Representative examples of A) Pd^II^‐ and B) Pd^0^‐catalysed cross‐coupling reactions of 5‐mercurated pyrimidine nucleosides. a) Methyl acrylate, Li_2_PdCl_4_, MeOH;^[23]^ b) iodobenzene, Pd(Ph_3_P)_4_, diglyme.^[45]^

The Heck coupling was originally described as a Pd^II^‐catalysed reaction between an organometallic compound and an alkene.^[19]^ The first step of the reaction, involving *in situ* transmetallation to yield the reactive organopalladium intermediate, was reported to proceed particularly smoothly with organomercury starting materials. While generation of the organopalladium intermediate by oxidative addition of Pd^0^ to an aryl halide is nowadays preferred for most applications, the easy availability of the organomercury starting material makes the original procedure still attractive for the C5‐functionalization of pyrimidine nucleosides (Scheme 1A).[^20^, ^21^, ^22^] Examples of substituents introduced by this approach range from simple vinylic and allylic groups[^23^, ^24^, ^25^, ^26^, ^27^, ^28^] to catalytically active side chains,[^29^, ^30^] linkers for further functionalization,[^31^, ^32^, ^33^, ^34^, ^35^, ^36^] carbohydrates[^37^, ^38^, ^39^] and metal complexes.[^40^, ^41^] Organic disulfides can also be used instead of alkenes, providing access to thioethers.[^42^, ^43^, ^44^] Finally, organomercury compounds also undergo a Pd^0^‐catalysed reaction with aryl halides, similar to the Negishi coupling of organozinc compounds (Scheme 1B).[^45^, ^46^]

### 3.2 Halodemercuration

**Scheme 2:**
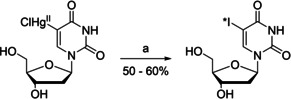
Iododemercuration of 5‐chloromercuri‐2′‐deoxyuridine. a) iodogen, Na*I, H_2_O; *I stands for ^123^I, ^125^I or ^131^I.[^48^, ^49^]

Sequential mercuration and halodemercuration provides access to halogenated aromatic compounds.^[2]^ In the field of nucleic acid chemistry this approach has found a niche application in the synthesis of radiolabelled nucleosides. Originally described soon after the first reports on organomercury nucleotides and nucleic acids,^[47]^ iododemercuration of 5‐chloromercuri‐2′‐deoxyuridine has later been refined into a facile and robust procedure for the preparation of ^123^I‐, ^125^I‐ and ^131^I‐labelled 5‐iodo‐2′‐deoxyuridine (Scheme 2).[^48^, ^49^] Remarkably, radiolabelling through iododemercuration (as well as bromodemercuration and reductive demercuration with sodium borotritiide) has been proven also on polymeric nucleic acids.^[47]^ Combined with the site‐selective mercuration of chemically synthesized oligonucleotides discussed above, halodemercuration should allow the preparation of oligonucleotides bearing a sterically conservative radiolabel at a predetermined site. Future studies will hopefully demonstrate the practical utility of such an approach.

## 4 Organomercury Nucleotides as Density Labels in Pycnographic Analysis

One of the earliest proposed applications of organomercury nucleotides relied on the sheer mass of the heavy mercury atom, as well as the applicability of 5‐mercuricytidine‐5′‐triphosphate as a substrate in enzymatic polymerization. The buoyant density of extensively mercurated DNA is considerably higher than that of native DNA and this difference can be exploited in CsCl density gradient centrifugation. Such DNA has been prepared through replication in permeabilized bacterial cells and found to band at a higher density than its unmodified counterpart.^[14]^ The use of organomercury nucleotides as pycnographic probes has not, however, gained widespread popularity.

## 5 The Potential of Organomercury Nucleotides as Markers in Electron Microscopy DNA Sequencing

The possibility of DNA sequencing by electron microscopy was first explored before the advent of Sanger sequencing and more modern methods.^[50]^ As the idea is again attracting attention, it is interesting to note that mercury was proposed as a heavy atom marker to facilitate the interpretation of DNA electron micrographs as early as 1974.^[7]^ The suggested procedure involved sequential treatment of the DNA with chloroacetaldehyde and mercuric acetate, resulting in near‐quantitative conversion of adenine and cytosine bases to covalently mercurated 1,*N* ^6^‐ethenoadenine and 3,*N* ^4^‐ethenocytosine bases (Figure 1F and G), respectively. To distinguish between mercurated adenine and cytosine bases, the DNA was first subjected to acid‐promoted depurination, after which the treatment described above afforded a sample having only the cytosine bases covalently mercurated. Finally, the positions of guanine and thymine bases would be inferred based on the complementarity rules of Watson‐Crick base pairing. The technological hurdles were too high to overcome at the time but more recently a very similar approach has proven successful.^[51]^ Instead of covalently mercurated 1,*N* ^6^‐ethenoadenine and 3,*N* ^4^‐ethenocytosine, methylmercury complex of 5‐mercaptouracil was used as the heavy atom marker, allowing undisrupted base pairing with adenine. The use of 5‐mercuricytosine would nicely complement this method as the labelled DNA could be prepared directly from native DNA by simple incubation with mercuric acetate.

## 6 Organomercury Nucleotides in X‐ray Crystallographic Structure Determination

Multiwavelength anomalous diffraction (MAD) is a phasing method used in the interpretation of X‐ray crystallographic data from biomacromolecules.^[52]^ The method requires incorporation of an anomalous scatterer, that is, a heavy atom, in the structure to be studied, ideally in such a way that the native three‐dimensional structure is not disturbed. In the case of nucleic acids, the easy introduction to C5 of pyrimidine bases makes mercury a particularly attractive candidate.^[53]^ The relatively wide major groove of DNA can accommodate a 5‐mercuri substituent with little disturbance of the double helix. In RNA the major groove is significantly narrower and mercuration should, hence, be confined near the ends of double‐helical regions.

## 7 Organomercury Nucleotides as Affinity Tags

The very high stability of Hg^II^‐thiol complexes can be harnessed for affinity chromatographic purification of covalently mercurated nucleic acids. Quantitative retention on thiol‐functionalized stationary phase, such as agarose, controlled‐pore glass or cellulose, is achieved through mercuration of as few as one base out of 200.^[16]^ Addition of a competing thiol, such as 2‐mercaptoethanol, in the mobile phase releases the mercurated nucleic acid from the stationary phase, allowing complete recovery. The method saw use in the early 1980s in the isolation of nascent nucleic acids from biological sources.[^54^, ^55^, ^56^, ^57^, ^58^] Mercuration was accomplished randomly throughout the sequence by incubating either permeabilized cells or isolated nuclei with 5‐mercuripyrimidine nucleoside triphosphates.

Covalent mercuration allows cellular nucleic acids to be not only isolated but also visualized within the cell.[^59^, ^60^, ^61^] Accordingly, nucleic acid probes of an appropriate sequence were mercurated by treatment with mercuric acetate and then allowed to hybridize sequence‐specifically with metaphase chromosomes or interphase nuclei. Finally, the cells were soaked with a thiol‐functionalized hapten ligand, either trinitrophenyl, biotinyl or fluorescyl. Visualization by fluorescence microscopy, either directly (in the case of fluorescyl) or after immunochemical amplification (in the case of trinitrophenyl and biotinyl) revealed localization of the ligands at the expected target sites.

## 8 Hg^II^‐Mediated Base Pairing of Organomercury Nucleobases

The concept of metal‐mediated base pairing[^62^, ^63^, ^64^, ^65^, ^66^, ^67^, ^68^, ^69^, ^70^] was first introduced in 1963 with mercury as the bridging metal ion^[71]^ and mercury still remains the most extensively studied metal in this context. Most of the research efforts have been directed at coordinative Hg^II^‐mediated base pairs, in particular the T‐Hg^II^‐T homo base pair.[^72^, ^73^, ^74^, ^75^] Such base pairs typically feature a dicoordinate bridging Hg^II^ and a linear coordination geometry although with artificial nucleobase surrogates higher coordination numbers have been reported as well.^[76]^ Various applications for coordinative Hg^II^‐mediated base pairing are under active development,^[63]^ ranging from sensors for Hg^II[77–81]^ to molecular wires.^[82]^ High affinity, rapid association and dissociation^[83]^ and responsiveness of nucleic acid secondary structure to subtle changes in the binding mode^[84]^ make Hg^II^‐mediated base pairs attractive components for the construction of DNA nanostructures.

The earliest mention of Hg^II^‐mediated base pairing of an organometallic nucleobase, albeit not termed as such, is the report from 1984 on formation of N3‐Hg^II^‐C5‐linked polymers of 5‐mercuriuridine.^[85]^ The idea was revisited in 1996 in a systematic ^1^H and ^199^Hg NMR study employing (1,3‐dimethyluracil‐5‐yl)mercury(II) as a model compound.^[86]^ N3‐methylation ruled out formation of the kind of coordination polymers observed previously with 5‐mercuriuridine, allowing Hg^II^‐mediated hetero base pairs with canonical nucleobases to be investigated. In line with previous studies on methylmercury,[^5^, ^87^] coordination at guanine N1 and thymine and uracil N3 with concomitant deprotonation of the donor atom was preferred.

More recently, Hg^II^‐mediated base pairing of organomercury nucleobases has been studied as a means to increase the hybridization affinity of oligonucleotide probes in biological media.^[11]^ These studies have involved recording concentration‐dependent ^1^H NMR spectra at monomer level and UV melting profiles at the oligomer level and, when applicable, the results have generally been in good agreement. In other words, high stability of an individual Hg^II^‐mediated base pair in solution translates into a high melting temperature of a double‐helical oligonucleotide incorporating the same base pair. Owing to the very rapid ligand exchange of Hg^II^, the NMR spectra typically only show one set of signals representing an average of all species in equilibrium. For the same reason, the UV denaturation and renaturation curves are superimposable even with heating and cooling rates typical of UV melting studies on unmodified oligonucleotide duplexes (e. g., 0.5 °C min^−1^).

### 8.1 Mononuclear Hg^II^‐mediated base pairs

**Figure 2:**
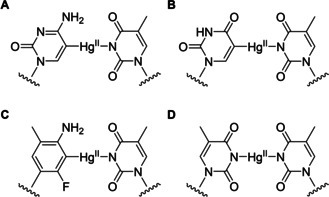
Mononuclear Hg^II^‐mediated base pairs formed by the organomercuric nucleobase analogues A) 5‐mercuricytosine, B) 5‐mercuriuracil and C) 3‐fluoro‐2‐mercuri‐6‐methylaniline with thymine are most likely isosteric with the T‐Hg^II^‐T base pair (D).

Flipping of a pyrimidine base from *anti* to *syn* conformation places its C5 in the position normally occupied by N3. Therefore, the C5‐Hg^II^‐N3‐linked base pairs of 5‐mercuricytosine^[88]^ and 5‐mercuriuracil^[89]^ with thymine (Figure 2A and B) are in all likelihood isosteric with the well‐documented N3‐Hg^II^‐N3‐linked base pair between two thymines (Figure 2D).[^72^, ^73^, ^74^, ^75^] A similar geometry seems likely also for the 3‐fluoro‐2‐mercuri‐6‐methylaniline‐thymine base pair (Figure 2C).^[8]^ Within oligonucleotides, all of these base pairs have a similar effect on the thermodynamic parameters of hybridization, namely increased (less negative) enthalpy and entropy owing to dehydration of the bridging Hg^II^ ion.[^73^, ^75^] Especially in the case of 3‐fluoro‐2‐mercuri‐6‐methylaniline, the latter effect more than compensates for the former, resulting in considerable duplex stabilization.^[8]^

### 8.2 Dinuclear Hg^II^‐mediated base pairs and triples

**Figure 3:**
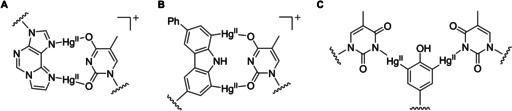
Dinuclear Hg^II^‐mediated base pairs and triples formed A) by 1,*N* ^6^‐ethenoadenine, B) 1,8‐dimercuri‐6‐phenyl‐1*H*‐carbazole and C) 2,6‐dimercuriphenol with thymine.

Covalent attachment of more than one Hg^II^ ion allows novel binding modes not resembling that of the T‐Hg^II^‐T base pair. Multinuclear organomercury nucleobases can be categorised as mono‐ or bifacial depending on the positioning of the Hg^II^ ions relative to each other. In monofacial bases, such as 1,8‐dimercuri‐6‐phenyl‐1*H*‐carbazole,^[10]^ the Hg^II^ bridges converge to bind to a single nucleobase, typically on its Watson–Crick face. NMR spectrometric or X‐ray crystallographic data on such base pairs is not available but high‐level DFT calculations have predicted remarkably similar structures for two dinuclear Hg^II^‐mediated base pairs with thymine, one coordinative (Figure 3A)[^90^, ^91^] and one organometallic (Figure 3B).^[10]^ In both of these base pairs, the two Hg^II^ ions coordinate to O2 and O4 of the thymine base. With the coordinative 1,*N* ^6^‐ethenoadenine‐Hg^II^_2_‐thymine base pair, more recent calculations suggest additional coordination to thymine N3 when this base pair is embedded within the base stack of a double helix.^[91]^ While very high stabilities have been reported for a number of multinuclear Ag^I^‐mediated base pairs,[^92^, ^93^, ^94^, ^95^, ^96^, ^97^, ^98^] corresponding results on these dinuclear Hg^II^‐mediated base pairs (organometallic or otherwise) were less impressive,[^10^, ^90^] perhaps owing to electrostatic repulsion between the Hg^II^ ions.

Bifacial multinuclear organomercury nucleobases are characterized by diverging Hg^II^ bridges binding to two other nucleobases. 2,6‐Dimercuriphenol, for example, forms stable dinuclear Hg^II^‐mediated base triples with adenine, cytosine and thymine.^[9]^ The latter, in particular, proved highly stabilizing in the middle of a homothymine*homoadenine⋅homothymine triple helix. As C2 and C6 of the 2,6‐dimercuriphenol nucleobase analogue are equivalent to C5 and N3 of pyrimidine nucleobases, base pairing at both Watson‐Crick and Hoogsteen faces most likely exhibits similar geometry as the mononuclear Hg^II^‐mediated base pairs discussed above (Figure 3C).

### 8.3 Hg^II^‐mediated base pairing in SNP genotyping

**Figure 4:**
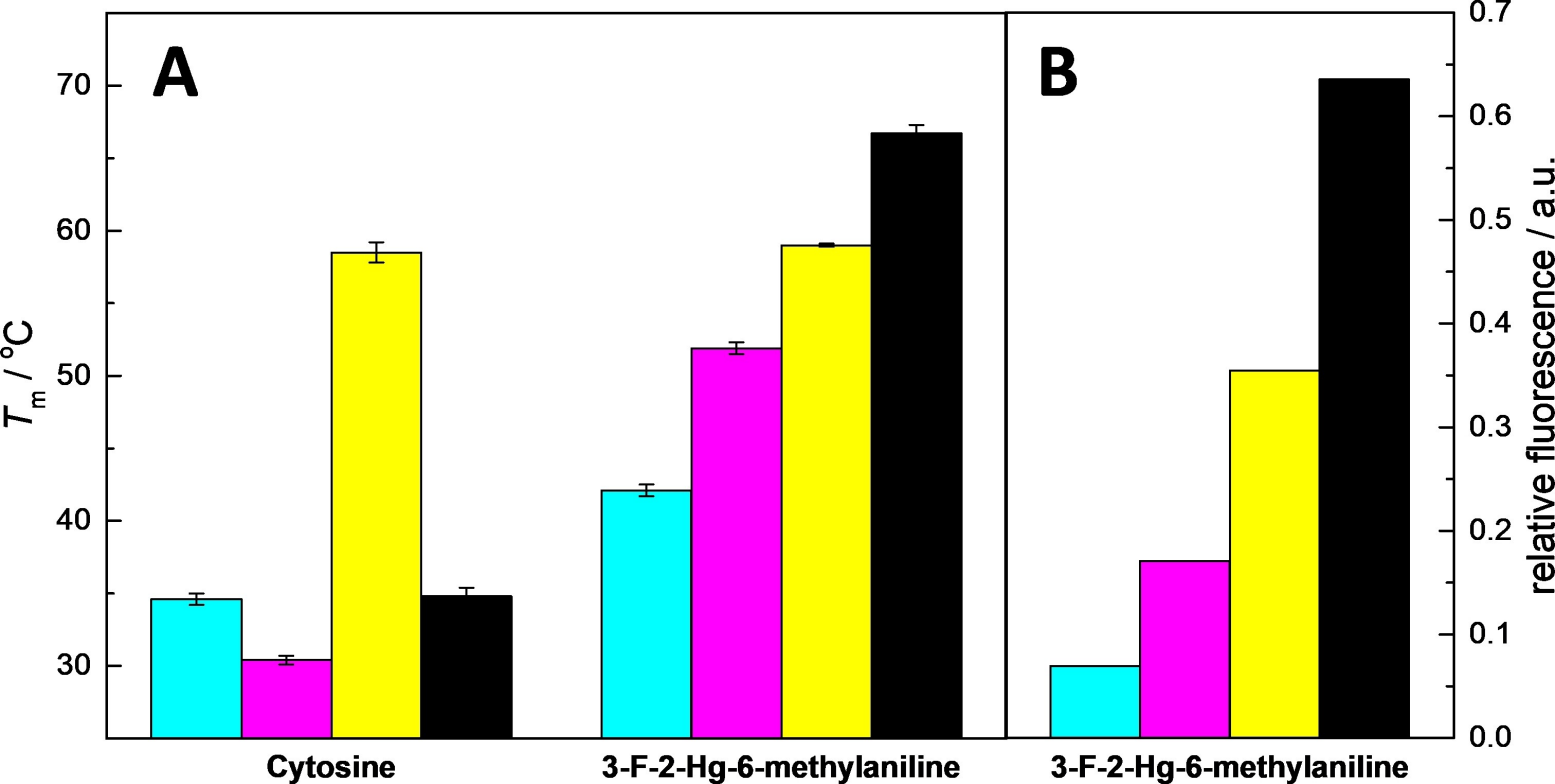
A) UV melting temperatures of short oligonucleotide duplexes incorporating a central base pair between either cytosine or 3‐fluoro‐2‐mercuri‐6‐methylaniline and adenine (cyan), cytosine (magenta), guanine (yellow) or thymine (black) and B) relative fluorescence emission intensity (*λ*_em_=520 nm, *T*=55 °C) of a molecular beacon featuring the same sequence in the recognition loop in the presence of respective target sequences.

Various methods for the detection of single nucleotide polymorphisms (SNPs) rely on differences in the hybridization affinities of oligonucleotide probes for the target sequence.[^99^, ^100^, ^101^, ^102^] Unfortunately, canonical Watson‐Crick base pairing is less than ideal for this approach. Although the matched base pair is usually much more stable than any of the mispairs, stabilities of the latter do not differ sufficiently from each other to allow reliable identification of the polymorphic nucleobase (Figure 4A). Metal‐mediated base pairing offers a way to overcome this limitation. Organomercury nucleobases exhibit very different base pairing preferences from their natural counterparts and 3‐fluoro‐2‐mercuri‐6‐methylaniline, in particular, stands out favourably for SNP genotyping. UV melting temperatures of short double‐helical oligonucleotides pairing this organomercury nucleobase with either adenine, cytosine, guanine or thymine all differed by at least 7 °C, enough for reliable identification of the variable base (Figure 4A).^[8]^ The identity of the base pairing partner was also clearly reflected in the ^19^F chemical shift of 3‐fluoro‐2‐mercuri‐6‐methylaniline. The feasibility of SNP genotyping based on metal‐mediated base pairing was recently demonstrated with a molecular beacon ‐type probe incorporating the 3‐fluoro‐2‐mercuri‐6‐methylaniline in the middle of the recognition loop.^[103]^ At appropriate temperature, stabilities of the different Hg^II^‐mediated base pairs translated into different hairpin‐heteroduplex equilibria and further into different fluorescence emission intensities (Figure 4B). Organomercury oligonucleotides, hence, hold great future potential as hybridization probes in SNP genotyping.

## 9 Summary and Outlook

Covalent mercuration of nucleic acids predates the efficient chemical synthesis of oligonucleotides. Organomercury nucleic acids were, hence, first employed in applications that do not rely on site‐specific mercuration, such as density labelling for pycnographic analysis or affinity tagging. These applications largely fell out of favour before oligonucleotide synthesis became mainstream, leaving their full potential unattained. Recently, synthetic oligonucleotides site‐specifically mercurated at predetermined natural or artificial hot spots have brought about a renaissance of organomercury nucleic acid chemistry. Potential new applications include the use of organomercury oligonucleotides as hybridization probes, notably in SNP genotyping. The scope of some established applications, such as radiolabelling through halodemercuration, could potentially be expanded to oligonucleotides. Finally, while coordinative Hg^II^‐mediated base pairing has already been harnessed in DNA nanotechnology, the use of organomercury oligonucleotides in this field remains an unexplored but interesting possibility.

## Conflict of interest

The authors declare no conflict of interest.

## Biographical Information

*After finishing his doctoral studies at the University of Turku, Finland, in 2005 under the supervision of Dr. Satu Mikkola, Tuomas Lönnberg joined the group of Professor Makoto Komiyama at the University of Tokyo as a JSPS Post‐Doctoral Fellow. In 2008, he returned to the University of Turku where he was appointed Assistant Professor of organic chemistry in 2016. His research interests include organometallic oligonucleotides and phosphate‐transfer reactions of nucleic acids*.

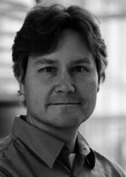

## Biographical Information

*Dattatraya Ukale received his M.Sc. from the University of Pune, India, in 2010. He then worked at Alkyl Amines Chemicals Ltd, the University of Pune and CSIR‐NCL, Pune in 2011, 2012 and 2016, respectively. Since 2017, he has been pursuing a doctoral degree under the supervision of Dr. Tuomas Lönnberg and will soon defend his thesis. His current research aims at development of organomercury oligonucleotides as high‐affinity probes for various natural nucleic acid targets*.